# Spindle cell lipoma of the tongue: First case report from the Gulf region and review of the literature

**DOI:** 10.1002/ccr3.8080

**Published:** 2023-10-17

**Authors:** Ahmed Hafez Mousa, Houriah Yasir Nukaly, Rawan Elwalid Ali Mohamed, Nagam AlShehabi, Rabbani Mahmoud Daoud, Abdelrahman Waleed Alsayed, Ramla Mohamed Farah Roble, Nigar Mehtiyeva, Farah Ennab, Temaa Alklani, Islam Khaled

**Affiliations:** ^1^ College of Medicine and Surgery Batterjee Medical College Jeddah Saudi Arabia; ^2^ Department of General Surgery Saudi German Hospitals Jeddah Saudi Arabia; ^3^ College of Medicine and Surgery International Medical University Kuala Lumpur Malaysia; ^4^ College of Medicine Mohammed Bin Rashid University of Medicine and Health Sciences Dubai United Arab Emirates; ^5^ School of Medicine Royal College of Surgeons of Ireland (RCSI) Busaiteen Kingdom of Bahrain; ^6^ Ahfad University for Women Omdurman Sudan; ^7^ Tbilisi State Medical University Tbilisi Georgia; ^8^ Faculty of Medicine Damascus University Damascus Syrian Arab Republic; ^9^ Department of Surgery, Faculty of Medicine Suez Canal University Ismailia Egypt

**Keywords:** lingual tumor, oral cavity lesion, spindle cell lipoma, tongue lipoma

## Abstract

Spindle cell lipoma is a histological variant of oral lipomas. In this report, we present the first case from the Gulf region with the most comprehensive literature review. Clinicians should consider rare tumors in the differential diagnoses of oral masses.

## Graphical abstract

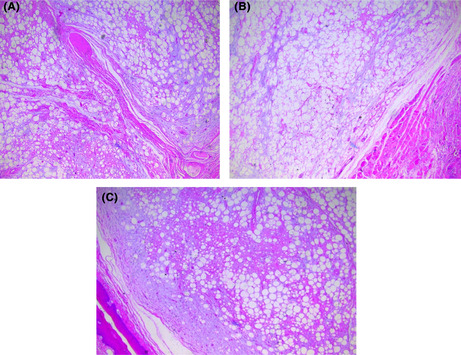

## 1 INTRODUCTION

Lipomas are prevalent benign soft tissue neoplasms of mesenchymal origin that develop in up to 20% of cases in the head and neck area.^1^ Oral lipoma is considered to account for 1% to 4% of all benign mouth lesions.^2^ Pleomorphic lipoma, angiolipoma, chondrolipoma, fibrolipoma, and spindle cell lipoma (SCL) are all histologic variations.^15^ Enzinger and Harvey were the first to report the SCL variant in 1975; morphologically, this variant has mature adipose tissue with bland spindle cells, myxoid stroma, ropey collagen bundles, and dispersed mast cells.^3^, ^15^ It was initially identified outside the oral cavity and tongue, and it remains an uncommon phenomenon across both locations.^7^ An asymptomatic, confined lump was the most common presenting complaint.^2^ However, tumors with equivalent form and odd presentations are becoming more frequent, rendering accurate diagnosis even more crucial.^12^ To be managed effectively, SCL must be pathologically distinguished from liposarcoma, other spindle cell neoplasms, and myxoid lesions.^4^, ^5^ Cytology, histology, immunohistochemistry, and cytogenetics, together with clinical presentation, are essential for determining the proper diagnosis of SCL.^4^, ^5^ The preferred therapy is surgical excision, which includes convenient removal from surrounding tissues.^5^ Lingual SCL has a benign clinical course with little chance of recurrence, although long‐term monitoring is required.^29^ The significance of this paper is to report a case of SCL in the tongue, shed some light on previously reported literature that is relatively sparse and portray the clinicopathological aspects of this unusual kind of oral Lipoma. The review of literature in our article entailed the search of PubMed as a primary database source to seek all published case reports in the available scientific literature. The key terms used were “Spindle Cell Lipoma” AND “Tongue Lesion” OR “Spindle Cell Tumor” AND “Lipoma of the Tongue.” All articles in the English language were included in our search. Certain variables such as demographic data, location of the tumor, diagnosis, treatment plans, and follow‐up were recorded and detailed in our results (Table 1).

**TABLE 1:** Summary of all previously reported cases of spindle cell lipomas (SCL) of the tongue.

Author	Year	Age (Years)	Gender	Clinical Presentation	Size (greatest diameter)	Laterality	Diagnosis	Treatment	Follow‐up
McDaniel et al.^6^	1984	52	M	—	—	—	Histology	Excision	96 months; NSR
Lombardi et al.^7^	1994	68	F	—	15 mm	Midline dorsum	Histology	Excision	‐
Domanski et al.^8^	1998	72	F	—	10 mm	Right	FNA + Histology	Excision	—
Dutt et al.^9^	1999	42	F	Gradually enlarging, painless swelling	30 mm	—	—	Excision	‐
Donen et al.^10^	2000	71	M	Painless swelling	Not mentioned	Left	Histology	Excision	25 months; NSR
Said‐Al‐Naief et al.^11^	2001	66	M	—	30 mm	—	Histology	Excision	12 months; NSR
Said‐Al‐Naief et al.^11^	2001	53	F	—	7 mm	—	Histology	Excision	24 months; NSR
Domanski et al.^12^	2001	71	F	—	10 mm	—	FNA + Histology	Excision	‐
Kaku et al.^13^	2003	75	M	Painful nodules	Right: 24 mm Left: 30 mm	Bilateral	Histology	Excision	—
Seiji et al.^14^	2003	56	M	Painless swelling	35 mm	Right	MRI with Contrast + Histology	Excision	11 months; NSR
Furlong et al.^15^	2004	—	—	—	—	—	—	—	—
Furlong et al.^15^	2004	—	—	—	—	—	—	—	—
Furlong et al.^15^	2004	—	—	—	—	—	—	—	—
Furlong et al.^15^	2004	—	—	—	—	—	—	—	—
Furlong et al.^15^	2004	—	—	—	—	—	—	—	—
Furlong et al.^15^	2004	—	—	—	—	—	—	—	—
Matsuura et al.^16^	2005	75	M	Painless mass	10 mm	Left	Histology	Excision	—
Billings et al.^17^	2006	45	M	Painless mass	9 mm	—	Histology	Excision	47 months; NSR
Billings et al.^17^	2006	67	M	Painless mass	10 mm	—	Histology	Excision	27 months; NSR
Billings et al.^17^	2006	31	F	Painless mass	3 mm	—	Histology	Excision	18 months; NSR
Billings et al.^17^	2006	75	F	Painless mass	5 mm	—	Histology	Excision	—
Shipchandler et al.^18^	2006	73	M	Slowly enlarging mass	20 mm	Left	Histology	Excision	—
Syed et al.^19^	2006	44	M	Painless mass and eating discomfort	8 mm	Midline	Histology	Excision	—
Imai et al.^20^	2008	72	M	Multiple painless masses bilaterally	Left: 12 and 15 mm Right: 5 mm	Bilateral	Histology	Excision	12 months; NSR
Júnior et al.^21^	2013	64	F	Progressively enlarging mass with dysphagia and talking difficulty	22 mm	Left	Ultrasound + Histology	Excision	—
Manor et al.^22^	2013	45	M	Painless mass	15 mm	Ventral	Histology	Excision	24 months; NSR
Seki et al.^23^	2015	78	M	Painless mass	7 mm	Right	Histology	Excision	14 months; NSR
Seki et al.^23^	2015	77	F	Painless mass	5 mm	Left	Histology	Excision	22 months; NSR
Lin et al.^24^	2015	63	F	Growing mass	30 mm + a smaller 8 mm	Right	Histology	Excision	—
Lau et al.^25^	2015	62	M	Incidental	10 mm	Left	Histology	Excision	115 months; NSR
Lau et al.^25^	2015	62	M	Painless mass	4 mm	Right	Histology	Excision	33 months; NSR
Lau et al.^25^	2015	61	F	Slowly growing painless mass	25 mm	Left	Histology	Excision	29 months; NSR
Lau et al.^25^	2015	80	M	Painless mass	13 mm	Left	Histology	Excision	13 months; NSR
Lau et al.^25^	2015	65	F	Slowly growing painless mass	2 mm	Right	Histology	Excision	118 months; NSR
Lau et al.^25^	2015	35	M	Painful mass	17 mm	Left	Histology	Excision	67 months; NSR
Lau et al.^25^	2015	47	F	Painless mass	—	Right	Histology	Excision	20 months; NSR
Lau et al.^25^	2015	47	M	Painless mass	5 mm	Left	Histology	Excision	11 months; NSR
Takahama Junior et al.^26^	2016	62	F	Painless mass	20 mm	Left	Histology	Excision	60 months; NSR
Minakawa et al.^27^	2016	68	M	Painless mass	42 mm and smaller 19 mm	Left	MRI + Biopsy+ Histology	Excision	12 months; NSR
Linares et al.^28^	2019	—	—	—	—	—	Histology	Excision	—
Shrestha et al.^29^	2021	7	M	Multiple growing painless masses causing difficulty speaking and mastication	Multiple, largest being 30 mm	Right + anterior	Histology	Excision	6 months; NSR
Okui et al.^30^	2023	79	M	Multiple painless masses	Left: 10 mm and Right: 6 mm	Bilateral	MRI + Histology	Interval Excision (3 mm margin in the second)	12 months; NSR
Okui et al.^30^	2023	86	M	Multiple painless masses	Left: 16 mm and Right: 20 mm	Bilateral	MRI + Histology	One‐stage Excision (3 mm margin)	12 months; NSR

Abbreviations: F, Female; M, male; mm, Millimeter; NSR, No sign of recurrence.

## 2 CASE PRESENTATION

A 33‐year‐old Saudi female presented to the general surgery clinic complaining of a swelling on the anterior aspect of the tongue. The mass was freely mobile with no impaired tongue mobility. She did not report any pain, dysphagia, dysphonia, dysgeusia, bleeding, dysarthria, or dyspnea. The mass was slowly growing over the course of several months. The patient denied having any other constitutional symptoms. Past medical and family history were both unremarkable. The rest of her history was noncontributory.

On physical examination, the patient's temperature was 37.3°C, blood pressure 121/84 mm Hg, and pulse 84 bpm with an SpO2 of 96% on room air. An approximately 20 × 10 mm submucosal soft, rubbery, and freely mobile mass over the anterior surface of the tongue was identified. Otherwise, the overlying mucosa was intact, the cervical lymph nodes were nonpalpable, and no other abnormalities were detected in the oral cavity. The patient is fit with BMI of 24.2 kg/m^2^. A provisional diagnosis of large tongue papilloma that is 30 mm in size was made.

Subsequently, she was scheduled for excisional biopsy and a series of laboratory tests were ordered. Beginning with a complete blood count (CBC), all parameters were within normal limits except for MCHC, which revealed a slight elevation of 34.70 g/dL. Additionally, further laboratory tests ordered included basic metabolic panel (BMP) whose components were all within reference range. The patient's blood group is O+ and negative for antibodies. Coagulation profile was also normal. Lastly, anti‐HCV, HBsAg, and HIV Ag/Ab were all found to be nonreactive.

**FIGURE 1:**
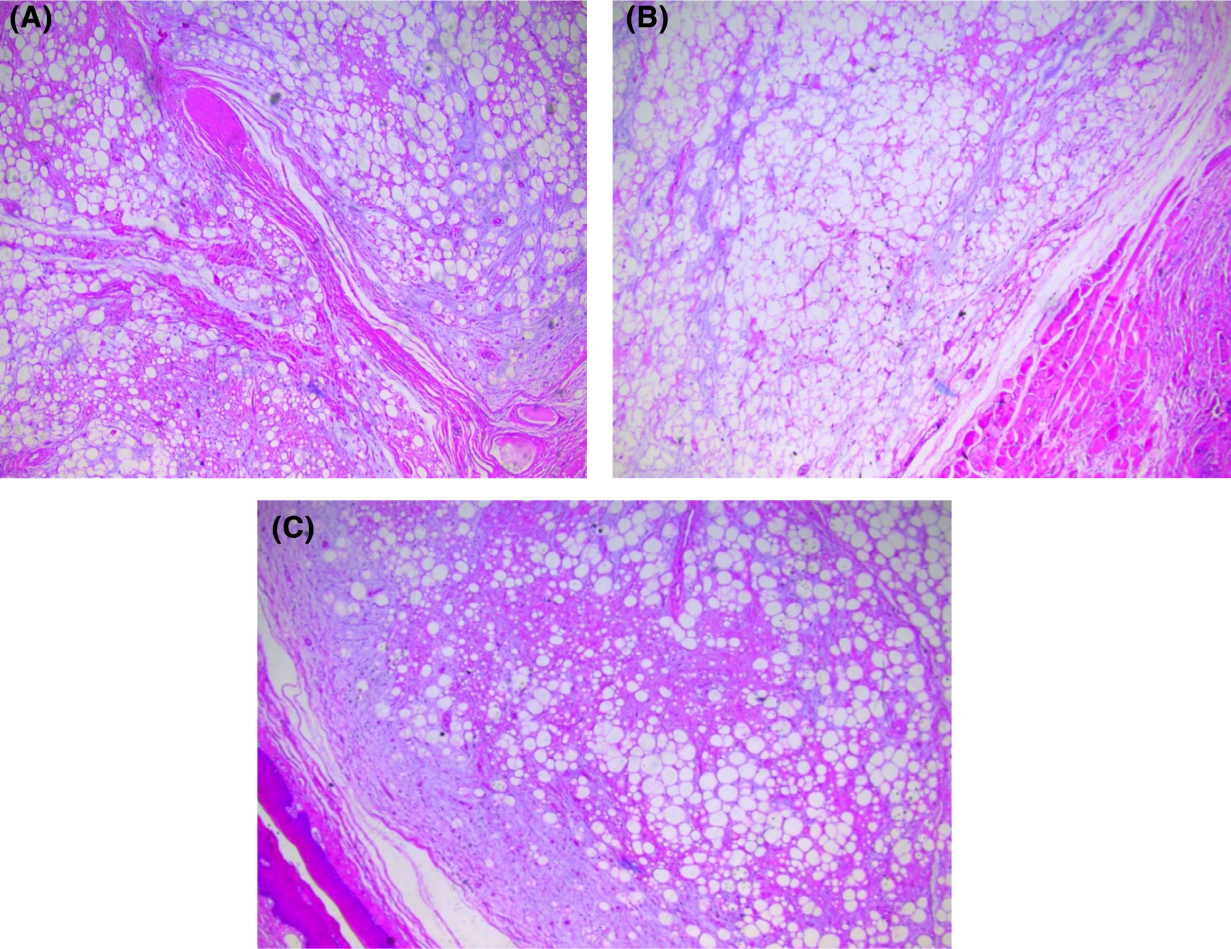
Histopathology of the resected specimen denoting (A) Spindle cell lipoma containing a mixture of adipocytes, bland spindle cells, and rubbery collagen fiber. (B) Benign adipocytic neoplasm showed muscle at the periphery. (C) Low power view of benign adipocytic neoplasm with bland spindle cells.

The patient underwent excisional biopsy under general anesthesia. The tumor was a polypoidal piece of grayish‐yellowish tissue measuring 15 × 10 mm. It was well‐encapsulated and excised in one cassette as shown in Figure 1. The patient was discharged on the same day without any postoperative complications with homecare instructions on Cefuroxime 500 mg PO (Oral) Tablet OD (Once Daily) for 14 days, Diclofenac Sodium 75 mg PO Capsule OD for 20 days, Miconazole 2% Oral Gel, Avalon (Azithromycin), and Povidone Iodine 10% Mouth Wash 135 mL. Postoperative follow‐up was uneventful, and the patient did not have any complaints regarding mastication or speech.

The histopathological findings revealed a marginally excised lipomatous tumor containing a mixture of adipocytes, bland spindle cells, and rubbery collagen fiber, consistent with SCL. Additionally, immunohistochemistry was positive for CD34 and absent for retinoblastoma 1 (Rb1). Other differentials such as well‐differentiated liposarcoma and lipoblastoma were excluded.

## 3 DISCUSSION

Lipomas are the most common soft tissue mesenchymal tumors.^15^, ^31^ The World Health Organization (WHO) classifies lipomatous tumors into several types—conventional lipoma, fibrolipoma, angiolipoma, spindle cell/pleomorphic lipoma, myxolipoma, chondroid lipoma, osteolipoma, myolipoma, lipomatosis, nerve lipomatosis, lipoblastoma/lipoblastomatosis, and hibernoma.^32^ Although lipomas are the most common mesenchymal tumors of soft tissue, they are rare in the oral region, accounting for no more than 2.2–4.4% of intraoral tumors.^15^, ^31^ The exact proportion of SCL among intraoral lipomas is a subject of controversy, as it has been suggested that SCL make up anywhere from 0 to 9.8% among intraoral lipoma cases.^24^ However, the largest study to date by Furlong et al.^15^ which analyzed 125 cases of lipomas in the oral and maxillofacial region, found SCL representing 59 (47%) of the cases.

The clinical presentations of lipomas are variable, with the most common picture being of slow‐growing painless masses that do not impair tongue mobility.^21^ However, some patients may present with a range of other symptoms, including dysphagia, dysphonia, dysgeusia, bleeding, or difficulty breathing and articulating speech.^25^, ^26^ The diagnosis of SCL of the tongue is usually based on histopathological examination, which reveals intersecting fascicles of bland spindle‐shaped cells and mature adipocytes, interspersed myxoid stroma, dispersed mast cells, and eosinophilic ropey collagen bundles.^3^, ^15^ Spindle cell lipoma and pleomorphic lipoma are currently considered by WHO^32^ to be the same entity, only differing by the presence of floret‐like or atypical stromal giant cells.^25^, ^33^ Immunohistochemical studies can also aid in the diagnosis of SCLs, although the marker profile, like CD34 positivity, is not specific to SCL and can be commonly seen in several other lipomatous and nonlipomatous soft tissue tumors.^10^ Typical SCLs show CD34‐positive cells; S‐100 protein‐positive adipocytes; S‐100 protein‐negative spindle cells; and MDM2, desmin, smooth muscle actin, CDK4‐negative cells.^25^, ^30^, ^34^ Differential diagnoses for SCL include lipoma with fibrosis, fibrolipoma, pleomorphic lipoma, atypical lipomatous tumor (ALT)/well‐differentiated liposarcoma (WDLPS), and solitary fibrous tumors (SFTs).^23^, ^30^

Upon reviewing 43 cases of SCLs of the tongue, we found that almost all the patients (except for one case by Shrestha et al.^29^) were adults, with an age range of 7–86 years, with a mean age of 61 years and median age of 64.5 years. Our case was for a 33‐year‐old female, which is the third youngest case reported and well below the reported median age. Our case revealed a male: female ratio of 1.57:1, which is in accordance with the reported male: female ratio ranging from 1.19:1 to 2.75:1.^15^, ^35^ Of note, our case reported no significant comorbidities or risk factors for the development of lipomas. The most common clinical presentation reported (24/30) was “painless mass” with only 2/30 cases presenting with painful masses.^13^, ^25^ In addition, 3/30 cases reported eating or speech difficulties.^19^, ^21^, ^29^ Of note, one case reported by Lau et al.^25^ presented as an incidental finding during another oral procedure. Our case similarly presented as a slowly growing painless mass over months.

While our case presented as an anterior lesion, the location of lingual SCL lesions reported in the literature varied between left border 12/27, right border 8/27, bilateral 3/27, midline 2/27, and ventral 1/27. The largest diameters of the SCL lesions reported in the literature ranged between 2 and 42 mm with a median of 12.5 mm and mean of 15.35 mm. Our patient presented with a mass measuring 30 mm in its largest diameter, which is relatively large compared with previously reported cases. Diagnosis in our case was established by histopathology and immunohistochemistry; however, cytogenetic studies were not done. Of note, two reported cases used fine‐needle aspiration (FNA) as part of their work‐up, which has shown considerable diagnostic efficacy.^8^, ^12^ In addition, five cases reported using additional diagnostic imaging (magnetic resonance imaging and ultrasound).^14^, ^21^, ^27^, ^30^ Reported cases relied on histopathologic examination and immunohistochemistry as a gold standard for diagnosis. While Mandahl et al.^36^ revealed a role for 16q13‐qter and 13q deletions in extra‐oral SCL/PL, none of the reviewed cases incorporated cytogenetic studies in their work‐up, and thus, the presence of this cytogenetic profile in lingual SCL cannot be ascertained.

All cases reported, as well as our case, were definitively treated with local excision. No operative complications or recurrences have been reported so far for lingual SCL, while one case of recurrence was reported by Fletcher et al.^37^ for extra‐oral SCL lesions. Follow‐up in reported cases ranged from 6 to 118 months, with a median follow‐up of 22 months and mean of 33.6 months. The ideal follow‐up duration and frequency remain largely unknown for lingual and generally oral SCL and merit future studies.

This study highlights that despite lingual SCL being initially considered a rare subtype of tongue neoplasm, there is an increasing number of reported lingual SCL cases among patients presenting with tongue masses. This emphasizes the relevance of accurate diagnosis of tongue lesions, whether by cytology, imaging, histopathology, immunohistochemistry, or cytogenetics as there is considerable diagnostic overlap among lipomatous and nonlipomatous pathologies of the tongue. In addition, the ideal follow‐up duration and frequency should be identified to minimize recurrences while ensuring patients' comfort.

Our study has certain limitations. First, conclusions from our literature review are limited as our review is derived from case reports rather than case–control, cohort studies, or experimental studies. Second, despite careful searches, we have probably not included all reported cases of lingual SCL. However, our review is the most complete review of all published lingual SCL cases to date.

## 4 CONCLUSION

To our knowledge, this is the first case of reported lingual SCL in the Gulf region. Our patient, a middle‐aged woman presented with a painless anterior tongue mass and was subsequently diagnosed with lingual SCL, a relatively rare tongue tumor despite her having no predisposing risk factors. Our literature review of 43 cases of SCL of the tongue highlights their variable presentation, the role of different diagnostic modalities (cytology, imaging, histopathology, immunohistochemistry, or cytogenetics), their relatively good outcomes, and their low risk of recurrence. Surgical excision remains the mainstay of treatment for these tumors. Overall, our case adds to the limited body of literature surrounding this rare tumor and emphasizes the importance of considering rare tumors in the differential diagnosis of oral masses.

## AUTHOR CONTRIBUTIONS

**Ahmed Hafez Mousa:** Conceptualization; data curation; formal analysis; writing – original draft. **Houriah Yasir Nukaly:** Data curation; methodology; writing – original draft. **Rawan Elwalid Ali Mohamed:** Data curation; writing – original draft. **Nagam AlShehabi:** Methodology; writing – original draft. **Rabbani Mahmoud Daoud:** Data curation; methodology; writing – original draft. **Abdelrahman Waleed Alsayed:** Data curation; writing – original draft. **Rawan Elwalid Ali Mohamed:** Writing – original draft. **Nigar Mehtiyeva:** Methodology; writing – original draft. **Farah Ennab:** Methodology; writing – review and editing. **Temaa Alklani:** Data curation; writing – review and editing. **Islam Khaled:** Conceptualization; project administration; supervision; writing – review and editing.

## FUNDING INFORMATION

This paper did not receive any source of funding to conduct.

## CONFLICT OF INTEREST STATEMENT

The authors declare that the research was conducted in the absence of any commercial or financial relationships that could be construed as a potential conflict of interest. The authors declare no conflict of interest.

## ETHICS STATEMENT

The study was conducted in accordance with the World Medical Association Declaration of Helsinki. Single case reports are exempted from ethical approval at our hospital.

## INFORMED CONSENT

Written informed consent was obtained from the patient for publication of this case report and accompanying images.

## Data Availability

All relevant data including all images supporting the findings of this report are included within the article.
